# ^18^F-FDG PET/CT response in a phase 1/2 trial of *nab-*paclitaxel plus gemcitabine for advanced pancreatic cancer

**DOI:** 10.1186/s40644-017-0125-5

**Published:** 2017-08-03

**Authors:** Ronald L. Korn, Daniel D. Von Hoff, Mitesh J. Borad, Markus F. Renschler, Desmond McGovern, R. Curtis Bay, Ramesh K. Ramanathan

**Affiliations:** 1 Imaging Endpoints Core Lab, 9700 N 91st St, B-200, Scottsdale, AZ 85258 USA; 2 Translational Genomics Research Institute and HonorHealth, 445 North Fifth St, Suite 600, Phoenix, AZ 85004 USA; 3 Mayo Clinic, 13400 E Shea Blvd, Scottsdale, AZ 85259 USA; 4 Celgene Corporation, 86 Morris Ave, Summit, NJ 07901 USA; 5 Department of Interdisciplinary Health Sciences, A. T. Still University, 5850 E Still Circle, Mesa, AZ 85206 USA

**Keywords:** Pancreatic cancer, *nab-*Paclitaxel, Gemcitabine, Phase 1/2 clinical trial, Positron emission tomography

## Abstract

**Background:**

Positron emission tomography (PET) is poised to become a useful imaging modality in staging and evaluating therapeutic responses in patients with metastatic pancreatic cancer (mPC). This analysis from a phase 1/2 study examined the utility of early PET imaging in patients with mPC treated with *nab*-paclitaxel plus gemcitabine.

**Methods:**

Tumors were measured by [^18^F]2-fluoro-2-deoxyglucose PET/computed tomography (CT) in patients who received *nab*-paclitaxel 100 (*n* = 13), 125 (*n* = 38), or 150 (*n* = 1) mg/m^2^ plus gemcitabine 1000 mg/m^2^ on days 1, 8, and 15 of a 28-day cycle. Lesion metabolic activity was evaluated at baseline and 6 and 12 weeks postbaseline.

**Results:**

Fifty-two patients had baseline and ≥1 follow-up PET scan. The median maximum standardized uptake values per pancreatic lesion in the *nab*-paclitaxel 100 mg/m^2^ and 125 mg/m^2^ cohorts were 5.1 and 6.5, respectively. Among patients who had a metabolic response by PET, those who received *nab*-paclitaxel 125 mg/m^2^ had a 4-month survival advantage over those who received 100 mg/m^2^. All patients in the *nab*-paclitaxel 125 mg/m^2^ cohort experienced an early complete metabolic response (CMR; 34%) or partial metabolic response (PMR; 66%). In the *nab*-paclitaxel 125 mg/m^2^ cohort, investigator-assessed objective response rates were 77% and 44% among patients with a CMR and PMR, respectively, with no correlation between PET and CT response (Spearman *r*_s_ = 0.22; *P* = 0.193). Patients in the *nab*-paclitaxel 125 mg/m^2^ cohort with a CMR experienced a significantly longer overall survival vs those with a PMR (median, 23.0 vs 11.2 months; *P* = 0.011), and a significant correlation was found between best percentage change in tumor burden by PET and survival: for each 1% decrease in PET score, the risk of death decreased by 2%.

**Conclusions:**

The majority of primary pancreatic tumors and their metastases were PET avid, and PET effectively measured changes in tumor metabolic activity at 6 and 12 weeks. These results support the antitumor activity of *nab*-paclitaxel 125 mg/m^2^ plus gemcitabine 1000 mg/m^2^ for treating mPC and the utility of PET for measuring treatment response. Treatment response by PET analysis may be considered when evaluating investigational agents in mPC.

**Trial registration:**

NCT00398086.

## Background

The use of positron emission tomography (PET) with [^18^F]2-fluoro-2-deoxyglucose as a tracer combined with computed tomography (^18^F-FDG/CT) to evaluate tumors has increased in recent years because of its high sensitivity and specificity. Currently, ^18^F-FDG/CT is used for diagnosing, initial staging, detecting recurrent disease, and evaluating response in various malignant tumors, including breast cancer, non-small cell lung cancer, colorectal cancer, esophageal cancer, head and neck cancer, cervical cancer, diffuse large B-cell lymphoma, and melanoma [1–10]. Primary pancreatic adenocarcinoma has also been shown to be FDG avid by PET/CT [11], and an association between metabolic response and survival has been demonstrated in patients with metastatic pancreatic cancer (mPC) [12], providing support for the feasibility of PET/CT for pancreatic tumors. Continued research may validate PET as a useful tool for measuring treatment response in this disease state.

The treatment of mPC is among the most challenging in clinical oncology, with a 5-year survival rate of only ≈ 3% [13, 14]. Since 1997, gemcitabine monotherapy has been a standard of care for treating mPC until recently, when the PRODIGE and MPACT trials demonstrated significant survival benefits with FOLFIRINOX (folinic acid, 5-fluorouracil, irinotecan, and oxaliplatin) and *nab*-paclitaxel plus gemcitabine, respectively, compared with gemcitabine monotherapy [15–17]. Based on the positive findings of the phase 3 MPACT trial, *nab*-paclitaxel plus gemcitabine was approved for the first-line treatment of mPC [17, 18]. Although these findings are encouraging, effective PC treatment remains a major challenge, and there is a need to identify those patients who are most likely to benefit from specific therapies.

At week 8 in the MPACT trial, ≈ 5 times more patients experienced a metabolic response by PET per criteria from the European Organisation for Research and Treatment of Cancer (EORTC) [19] than an objective response by CT per Response Evaluation Criteria In Solid Tumors (RECIST) [20, 21], and patients with a tumor response identified by either modality had a median overall survival (OS) of >10 months [12]. The initial findings on the utility of PET/CT to assess response with *nab*-paclitaxel plus gemcitabine in a phase 1/2 study of patients with mPC were previously reported [22]. The preliminary data demonstrated that a complete loss of FDG metabolic activity was associated with an improved OS with *nab*-paclitaxel plus gemcitabine [22]. Furthermore, assessment of treatment response by PET was more sensitive than by CT; for all patients, the median decrease in metabolic activity at 12 weeks was 79% (*n* = 55; by ^18^F-FDG PET scan using EORTC criteria), whereas the overall response rate was 46% (*n* = 67; by CT scan using RECIST v1.0) [22]. Here we report the final analysis of the PET/CT data from the phase 1/2 trial and a more detailed analysis of the ^18^F-FDG PET/CT data in measuring efficacy outcomes in patients with mPC who received *nab*-paclitaxel 125 or 100 mg/m^2^ plus gemcitabine.

## Methods

This open-label study, consisting of a phase 1 dose-finding portion and an expanded phase 2 portion at the maximum tolerated dose, was conducted at 4 centers in the United States in accordance with the Declaration of Helsinki and Good Clinical Practice Guidelines of the International Conference on Harmonization. Written informed consent was obtained from all patients before they entered the study. Details of the patients and methods in this study were reported previously [22]. Briefly, patients aged ≥18 years who had histologically or cytologically confirmed mPC with measurable disease by CT scan, as defined by the RECIST v1.0 guidelines, and no previous treatment for metastatic disease received *nab*-paclitaxel (100, 125, or 150 mg/m^2^) followed by gemcitabine (1000 mg/m^2^)—both administered intravenously on days 1, 8, and 15 every 28 days. Sixty-seven patients were enrolled in the trial: 20, 44, and 3 patients received 100, 125, and 150 mg/m^2^ *nab*-paclitaxel, respectively. The maximum tolerated dose was established as 125 mg/m^2^ of *nab*-paclitaxel plus 1000 mg/m^2^ of gemcitabine once a week for 3 weeks every 28 days, which was the dose and schedule used in the phase 3 MPACT trial [17, 22]. This regimen is also the US Food and Drug Administration–approved dose and schedule for the treatment of mPC according to the prescribing information [18].

### ^18^F-FDG PET/CT acquisition and scan lesion evaluation

^18^F-FDG PET/CT scans of target and nontarget lesions were taken at baseline and at 6 and/or 12 weeks postbaseline. Patients underwent at least a 4-h fast prior to each scan. Patients were injected with a mean 5.5 × 10^11^ mBq ^18^F-FDG (range, 3.3 × 10^11^–10.4 × 10^11^ mBq) based upon body mass 0.44 × 10^10^ to 1.5 × 10^10^ mBq/kg. All patients’ fasting glucose values were within an acceptable range (< 200 mg/dL). The average uptake period was 66 min (range, 50 to 110 min; 88% of all scans were conducted at an uptake period of 50 to 85 min). Each patient also underwent a low-dose CT scan for attenuation correction followed by a whole-body (orbitomeatal line to mid thighs) PET emission scan. The PET images were acquired in 2D and 3D modes per site standards (2–5 min per bed position) and then iteratively reconstructed with z-axis postprocessing filtering and corrected for attenuation using the low-dose CT scan series.

#### Baseline

Target lesions were chosen based on size and location. No more than 5 lesions in any 1 organ system and no more than 10 lesions in total were chosen for analysis of standardized uptake values (SUVs). The maximum SUV (SUV_max_) of all target lesions within a patient was then summed and served as a measure of tumor burden at the baseline time point, referred to as SUV_max_ Total. Note that SUV_max_ has been commonly used, particularly at the time this study was conducted, to evaluate tumor burden and change in tumor burden by PET [3–7, 12, 23]. Nontarget lesions were noted, and the anatomical location of both target and nontarget lesions was recorded.

#### Follow-up

Scans of target and nontarget lesions were performed at weeks 6 and 12 postbaseline. The SUV_max_ of all target lesions was then summed, and the total served as a measure of tumor burden for that particular scan (SUV_max_ Total). The images were then evaluated for the presence of new lesions on the ^18^F-FDG PET/CT fused images as appropriate.

In addition to the confirmatory CT scans following the PET imaging at 6 and 12 weeks, contrast-enhanced diagnostic-quality spiral CT scans were performed separately at baseline and every 4 weeks to evaluate tumor response to therapy per RECIST. The diagnostic CT scans required the evaluation of the chest, abdomen, and pelvis using the kVp, mAs, and slice thickness ≤ 5 mm to perform lesion assessments according to RECIST guidelines. Comparisons of PET/CT and diagnostic-quality spiral CT scans as markers of efficacy at specific time points were not feasible because of the difference in timing of the scans.

### Image analysis

De-identified images were interpreted by a single reader (RLK). Images were inspected for quality, body coverage, and dose infiltration at the injection site and for other factors that could have altered quantitative analysis. The images were interpreted to determine treatment response using the PET/CT and image-fusion ^18^F-FDG PET/CT data sets. The locations of the metastatic lesions were noted, and SUVs were determined when appropriate. The SUV activity in normal tissue of the liver and mediastinum was recorded. ^18^F-FDG activity was considered to indicate malignancy when the ^18^F-FDG activity was focal, was greater than the background ^18^F-FDG metabolism, and had a corresponding abnormality on CT. Target lesions were selected on baseline PET/CT scans without guidance from follow-up scans. Up to 5 lesions were selected for target lesion SUV_max_ analysis from visual inspection of the lesions with the greatest FDG uptake. Both primary pancreatic and metastatic lesions were included. All other hypermetabolic tumor lesions were considered nontarget lesions. Subsequently, SUV_max_ measurements of the target lesions were assessed on follow-up PET/CT scans. In the case of resolved hypermetabolic activity, the target lesions were given an SUV_max_ value of 0. Nontarget lesions were followed qualitatively for 1) complete resolution of FDG activity, 2) significant increase of FDG uptake compared to baseline scans, or 3) neither resolution nor significant increase (ie, metabolically stable) of FDG activity. Finally, any FDG focus that was consistent with malignant uptake that was not present previously was declared a new lesion if, in the opinion of the reader, it was consistent with malignant uptake. In order to assign time-point response, the sum of the SUV_max_ for target lesions was used to assess interval change from baseline along with a determination of new lesion development and change in nontarget lesion FDG behavior for final response assignment (Table 1).

**Table 1:** EORTC criteria for determining tumor response by PET [19]

Classification	Description
Progressive metabolic disease	• An increase in ^18^F-FDG tumor SUV of >25% within the tumor region defined on the baseline scan • Visible increase in the extent of ^18^F-FDG tumor uptake (>20% in the longest dimension) • The appearance of new ^18^F-FDG uptake in metastatic lesions
Stable metabolic disease	• An increase in tumor ^18^F-FDG SUV of <25% • A decrease of <15% and no visible increase in the extent of ^18^F-FDG tumor uptake (>20% in the longest dimension)
Partial metabolic response^a^	• A decrease in tumor uptake of ^18^F-FDG >25% after >1 cycle of treatment
Complete metabolic response	• Complete resolution of ^18^F-FDG uptake within the tumor volume so that it is indistinguishable from surrounding normal tissue

*EORTC* European Organisation for Research and Treatment of Cancer, ^18^ *F-FDG* [^18^F]2-fluoro-2-deoxyglucose, *PET* positron emission tomography, *SUV* standardized uptake value

^a^EORTC criteria also define a decrease in tumor uptake of ^18^F-FDG of ≥15% to 25% after 1 cycle of treatment as a partial metabolic response; however, the more stringent 25% threshold was applied to all patients in this study

### SUVs

Standardized uptake values were determined using an SUV function integrated into a GE Advantage Window Workstation. In order to obtain the SUV measurement, a 1.5-cm region of interest (ROI) was deposited on the image slice that had the most intense ^18^F-FDG activity determined by visual inspection. By definition, an SUV is a reflection of the amount of ^18^F-FDG activity in an ROI per the following formula:

$$ SUV\kern0.5em =\kern0.5em \left[\mathrm{activity}/\mathrm{mL}\ \mathrm{tissue}\ \left(\mathrm{decay}\hbox{-} \mathrm{corrected}\right)\right]/\left(\mathrm{injected}\ \mathrm{dose}/\mathrm{body}\ \mathrm{weight}\right). $$

### Calculation of % tumor response on ^18^F-FDG PET/CT

The percentage of change in SUV activity was calculated using the following formula:

$$ \begin{array}{l}\kern15em \%\mathrm{Change}\ {SUV}_{\max}\mathrm{Total}=\\ {}\left[{SUV}_{\max}\mathrm{Total}\ \left(\mathrm{current}\ \mathrm{scan}\right)\left]\kern0.5em -\kern0.5em \right[{SUV}_{\max}\mathrm{Total}\ \left(\mathrm{baseline}\ \mathrm{scan}\right)\right]\kern0.5em \times \kern0.5em 100\\ {}\kern15em \left[{SUV}_{\max}\mathrm{Total}\ \left(\mathrm{baseline}\right)\right]\end{array} $$

The metabolic response category on the follow-up scans was then determined based on the EORTC criteria for PET response (Table 1) [19].

### Statistical analysis

The characteristics of the target lesions were analyzed using standard descriptive statistics. Statistical analyses were carried out using SPSS 19 software. The best ^18^F-FDG PET/CT response rate for each patient, measured as the percentage of change in SUV_max_ Total at follow-up compared with baseline, was calculated and classified per EORTC criteria (Table 1); note that the more stringent threshold of a ≥ 25% decrease was always used to define partial response because even the first postbaseline scan was at week 6, which was considered to be >1 cycle of treatment (see Table).

The relationship between the best PET response and OS was tested, using a log-rank test, in the group with a complete metabolic response (CMR, defined in Table 1) vs the non-CMR group (all had a partial metabolic response [PMR]) in the *nab*-paclitaxel 125 mg/m^2^ dose cohort. A Cox proportional hazards model was used to determine whether the best percentage of change in PET was associated with OS. A nonparametric rank-correlation test was used to determine whether a correlation existed between the best percentage of PET and radiographic (using CT) changes; the data were summarized by Spearman rank-correlation coefficient. The relationship of CT and PET response was also evaluated by comparing rates of objective response in patients who had a CMR vs a PMR (CT and PET responses evaluated as categorical variables). A *P* value ≤ 0.05 was considered statistically significant.

## Results

Of the 67 enrolled patients, 61 had a baseline ^18^F-FDG PET/CT scan, and 52 patients had both a baseline ^18^F-FDG PET/CT scan and at least 1 follow-up ^18^F-FDG PET/CT scan at 6 weeks, 12 weeks, or both. Of the 20 treated patients in the *nab*-paclitaxel 100 mg/m^2^ cohort, 13 had a baseline and at least 1 follow-up ^18^F-FDG PET/CT scan at 6 weeks, 12 weeks, or both. Of the 44 treated patients in the *nab*-paclitaxel 125 mg/m^2^ cohort, 38 had a baseline and at least 1 follow-up ^18^F-FDG PET/CT scan at 6 weeks, 12 weeks, or both. Lack of follow-up scans was primarily due to disease progression, patient choice, or logistical/monetary reasons. Of 13 patients in the *nab*-paclitaxel 100 mg/m^2^ cohort who had at least 1 follow-up ^18^F-FDG PET/CT scan, 12 had an ^18^F-FDG PET/CT scan at 6 weeks, and 12 had an ^18^F-FDG PET/CT scan at 12 weeks. Of 38 patients in the *nab*-paclitaxel 125 mg/m^2^ cohort who had at least 1 follow-up ^18^F-FDG PET/CT scan, 37 had an ^18^F-FDG PET/CT scan at 6 weeks, and 34 had an ^18^F-FDG PET/CT scan at 12 weeks. The median age of all patients was 61 years and of those in the *nab*-paclitaxel 100 and 125 mg/m^2^ cohorts was 57 and 61 years, respectively (Table 2).

**Table 2:** Selected baseline patient characteristics for patients with a baseline and ≥1 follow-up PET scan

Characteristic	*nab*-Paclitaxel Dose^a^		
	100 mg/m^2^ (*n* = 13)	125 mg/m^2^ (*n* = 38)	All dose levels (*n* = 52)
Age, median (range), years	57.0 (30–79)	61.0 (28–76)	61.0 (28–79)
Male sex, n (%)	7 (54)	17 (45)	24 (46)
ECOG PS, n (%)			
0	6 (46)	21 (55)	28 (54)
1	7 (54)	17 (45)	24 (46)
CA 19–9 at baseline, median (range), units/mL	*n* = 12 1220.1 (16.8–180,062.0)	*n* = 37 880.8 (1.1–96,990.0)	*n* = 50 1062.6 (1.1–180,062.0)

^a^1 patient received *nab*-paclitaxel 150 mg/m^2^ and their baseline characteristics are included in the “All dose levels” column

*CA 19–9* carbohydrate antigen 19–9, *ECOG PS* Eastern Cooperative Oncology Group performance status, *PET* positron emission tomography

### Distribution of lesions on ^18^F-FDG PET/CT

The distribution and baseline SUV_max_ of lesions from patients enrolled in this study are summarized in Table 3. Among all patients, primary pancreatic (*n* = 43) lesions were the most common target lesions examined. The mean SUV_max_ of pancreatic lesions was 6.9 (median, 6.4; range, 1.5–17.5). The most common sites of metastases were liver (*n* = 37), with an average SUV_max_ of 7.6 (median, 6.6; range, 2.2–15.7); peritoneum (*n* = 28), with an average SUV_max_ of 5.9 (median, 5.3; range, 0.6–20.7); and mediastinal lymph nodes (*n* = 18), with an average SUV_max_ of 4.5 (median, 4.0; range, 1.5–11.3). These findings were generally consistent between the 2 dose cohorts, with liver and peritoneum being the most common sites of metastases. Metastatic lesions were identified by PET/CT in most anatomical sites, with the exception of the brain and kidney. This distribution of involved sites is consistent with the known spread of PC metastasis by both lymphatic and hematogeneous routes.

**Table 3:** Lesions at baseline^a^

Lesion Location	*nab*-Paclitaxel 100 mg/m^2^	*nab*-Paclitaxel 125 mg/m^2^	All dose levels		
	(*n* = 13)	(*n* = 38)	(*n* = 51)	Baseline SUV_max_	
	*n* (%)	*n* (%)	*n* (%)	Mean (St Dev)	Median
Pancreas	11 (85)	32 (84)	43 (84)	6.9 (3.6)	6.4
Liver	8 (62)	29 (76)	37 (73)	7.6 (3.4)	6.6
Peritoneum	4 (31)	24 (63)	28 (55)	5.9 (3.9)	5.3
Mediastinal nodes	3 (23)	15 (39)	18 (35)	4.5 (2.9)	4.0
Lung	3 (23)	11 (29)	14 (27)	2.6 (3.0)	2.1
Pelvic nodes	2 (15)	7 (18)	9 (18)	6.8 (2.7)	7.6
Hilar nodes	2 (15)	7 (18)	9 (18)	3.8 (1.8)	4.1
Neck nodes	1 (8)	5 (13)	6 (12)	6.7 (4.8)	5.1
Omentum/mesentery	1 (8)	5 (13)	6 (12)	6.8 (2.2)	6.6
Pleura	4 (31)	2 (5)	6 (12)	2.8 (2.0)	1.8
Bone	1 (8)	2 (5)	3 (6)	5.0 (1.4)	4.2
Adrenal glands	0	3 (8)	3 (6)	2.7 (1.4)	2.1
Spleen	0	2 (5)	2 (4)	3.1 (0.3)	3.1
Skin	2 (15)	0	2 (4)	5.9 (1.8)	5.9
Muscle	0	2 (5)	2 (4)	5.3 (1.1)	5.3
Other	0	0	0	Not Applicable	
Brain	0	0	0	Not Applicable	
Kidneys	0	0	0	Not Applicable	

^a^Based on a nominal alpha of .05 (2-tailed) and using Fisher exact tests, the 2 groups differed significantly on number of lesions in only 1 site: the pleura (*P* = 0.031)

### Individual lesion analysis

**Fig. 1:**
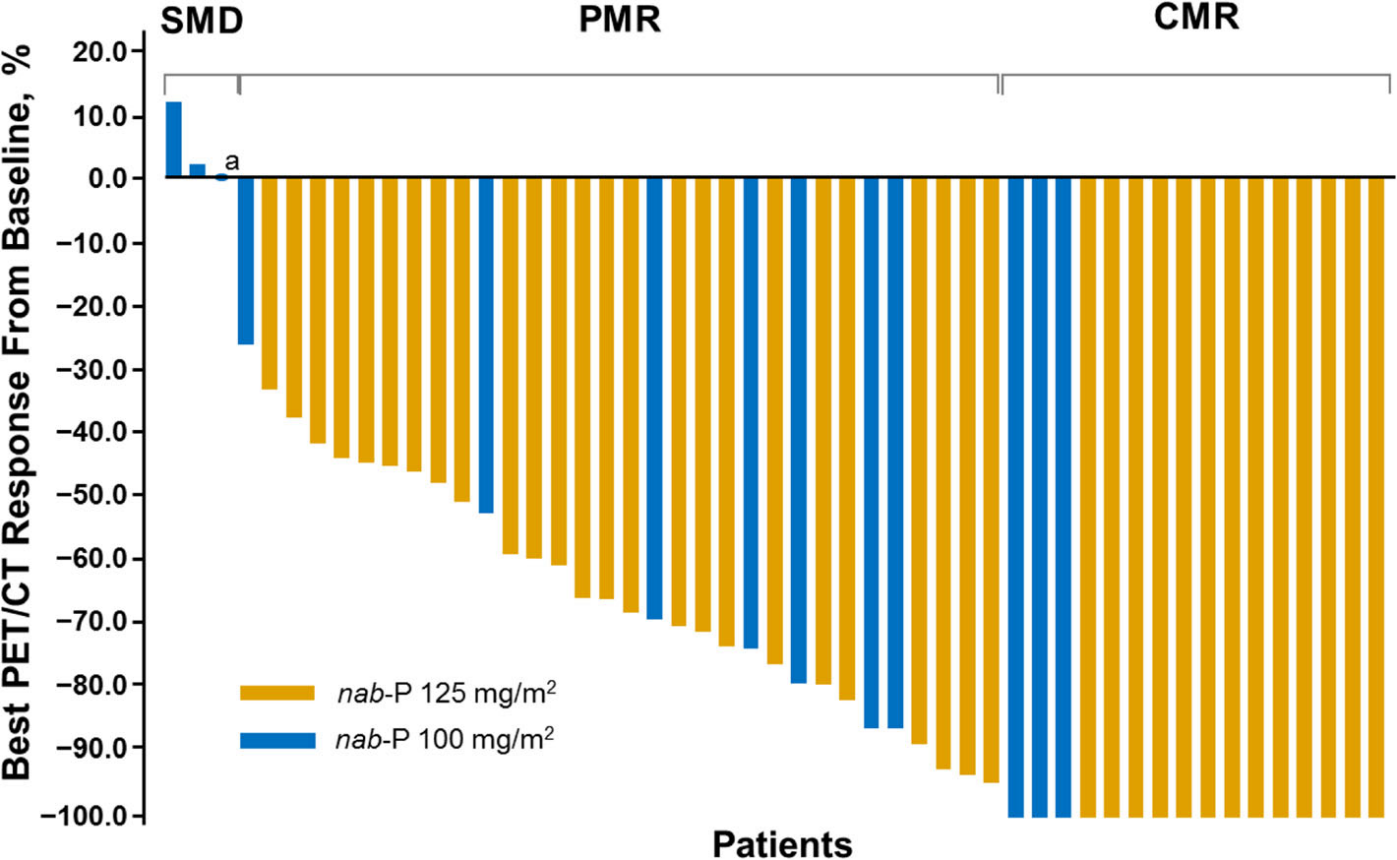
Waterfall plot of best responses by ^18^F-FDG PET/CT. *CMR*, complete metabolic response; *CT*, computed tomography; *nab-P*, nab-paclitaxel; *PET*, positron emission tomography; *PMR*, partial metabolic response; *SMD*, stable metabolic disease. ^a^ The blue circle represents 0% best response from a single patient in the nab-P 100 mg/m^2^ cohort

A total of 42 and 154 lesions were evaluated on the ^18^F-FDG PET/CT scans for patients in the *nab*-paclitaxel 100 and 125 mg/m^2^ cohorts, respectively. The median numbers of target lesions per patient to be scanned by ^18^F-FDG PET/CT at baseline were 3.0 and 4.0 in patients in the *nab*-paclitaxel 100 and 125 mg/m^2^ cohorts, respectively. The best PET responses from baseline in patients in the *nab*-paclitaxel 100 and 125 mg/m^2^ cohorts are shown in Fig. 1. Among patients in the *nab*-paclitaxel 100 mg/m^2^ cohort, the median SUV_max_ per lesion was 5.1 (quartiles 1–3 range, 3.4–11.2). Among patients in the *nab*-paclitaxel 125 mg/m^2^ cohort, the median SUV_max_ per lesion was 6.5 (quartiles 1–3 range, 3.9–9.5).

### ^18^F-FDG PET/CT metabolic response

A summary of the ^18^F-FDG response by dose cohort is presented in Table 4. Of the 52 evaluable patients, 17 (33%) had an EORTC-defined CMR, 32 (62%) had a PMR, and 3 (6%) had stable metabolic disease. No patients had progressive disease based on PET during this testing interval up to 12 weeks. Patients in the *nab*-paclitaxel 100 mg/m^2^ cohort (*n* = 13) experienced an EORTC-defined CMR (3 [23%]), a PMR (7 [54%]), or stable metabolic disease (3 [23%]). Patients in the *nab*-paclitaxel 125 mg/m^2^ cohort (*n* = 38) had either an EORTC-defined CMR (13 [34%]) or a PMR (25 [66%]).

**Table 4:** ^18^F-FDG PET/CT best response

*nab*-Paclitaxel Dose Cohort^a^, n (%)	CMR	PMR	SMD	PMD
100 mg/m^2^ (*n* = 13)	3 (23)	7 (54)	3 (23)	0
125 mg/m^2^ (*n* = 38)	13 (34)	25 (66)	0	0

^a^1 patient received *nab*-paclitaxel 150 mg/m^2^ and their best response by ^18^F-FDG PET/CT was CMR

*CMR* complete metabolic response, *CT* computed tomography, ^*18*^ *F-FDG* [^18^F]2-fluoro-2-deoxyglucose, *PET* positron emission tomography, *PMD* progressive metabolic disease, *PMR* partial metabolic response, *SMD* stable metabolic disease

Among patients in the *nab*-paclitaxel 100 mg/m^2^ cohort with a follow-up ^18^F-FDG PET/CT scan at 6 and 12 weeks (*n* = 11), 6 (55%) had a decrease, 4 (36%) had an increase, and 1 (9%) had no change (either no change or change <1%) in SUV_max_ from week 6 to week 12. Among patients in the *nab*-paclitaxel 125 mg/m^2^ cohort with a follow-up ^18^F-FDG PET/CT scan at 6 and 12 weeks (*n* = 33), 19 (58%) had a decrease, 7 (21%) had an increase, and 7 (21%) had no change (either no change or change <1%) in SUV_max_ from week 6 to week 12.

### ^18^F-FDG PET/CT response and median overall survival

**Fig. 2:**
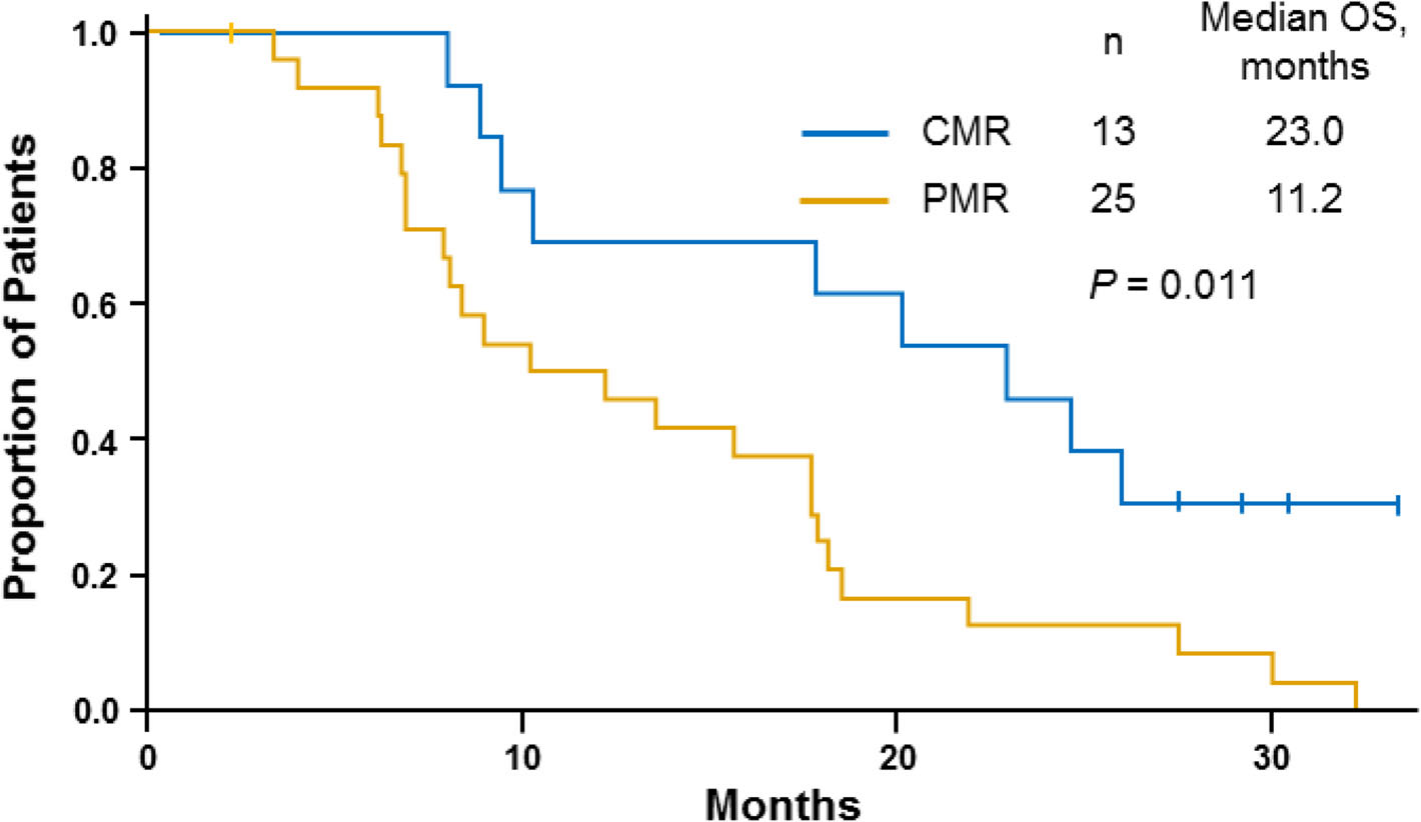
Overall survival by PET metabolic response among patients in the *nab*-paclitaxel 125 mg/m^2^ cohort. *CMR*, complete metabolic response; *OS*, overall survival; *PET*, positron emission tomography; *PMR*, partial metabolic response

Among patients who achieved a CMR or PMR by PET, a survival difference of ≈ 4 months was observed in favor of the *nab*-paclitaxel 125 mg/m^2^ cohort vs the 100 mg/m^2^ cohort (median, 15.6 vs 11.4 months, respectively; Table 5). In the *nab*-paclitaxel 125 mg/m^2^ cohort, the OS in patients with a CMR was significantly longer than that of patients with a PMR (median, 23.0 vs 11.2 months; *P* = 0.011; Fig. 2). Within this cohort, a significant correlation was observed between best percentage of change in tumor burden by PET, evaluated as a continuous variable, and OS; namely, for each 1% decrease in PET SUV, the risk of death decreased by 2% (hazard ratio 0.98; 95% CI, 0.965–0.995; *P* = 0.010).

**Table 5:** Overall survival by *nab*-paclitaxel cohort and response

	*nab*-Paclitaxel Dose	
	100 mg/m^2^	125 mg/m^2^
All PET-evaluable patients, n	13	38
Median OS, months	10.9	15.6
Patients with a CMR or PMR, n	10	38
Median OS, months	11.4	15.6
All patients, n	20	44
Median OS, months	9.3	12.2^a^

^a^Data previously published [22]

*CMR* complete metabolic response, *OS* overall survival, *PET* positron emission tomography, *PMR* partial metabolic response

### Radiographic response by CT scan and relationship with PET

**Fig. 3:**
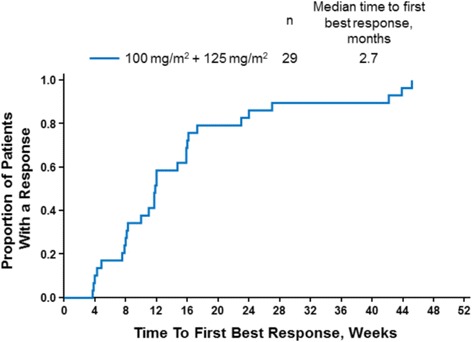
Time to best response by CT in the 125 and 100 mg/m^2^ *nab*-paclitaxel cohorts. *CT*, computed tomography

Among patients in the *nab*-paclitaxel 125 mg/m^2^ cohort, the investigator-assessed objective CT response rates by diagnostic (spiral) CT per RECIST v1.0 were 77% in patients with a CMR vs 44% in patients with a PMR (both evaluated as categorical variables; *P* = 0.053); 21 of 38 patients had a partial response, and 8 of 38 patients had stable disease for ≥16 weeks by CT scan. The every-4-week response rates (per CT) over 28 weeks are reported in Table 6. The median time to best response by CT, as determined by the investigator, was 2.7 months collectively in patients in both the *nab*-paclitaxel 125 and 100 mg/m^2^ cohorts (Fig. 3). No significant correlation was observed between best percentage of change in tumor burden by ^18^F-FDG PET/CT and radiographic measurement by CT scan (Spearman analysis; *r*_s_ = 0.22; *P* = 0.193).

**Table 6:** Every-4-week spiral CT tumor response rates in the 125 mg/m^2^ *nab*-paclitaxel cohort over 28 weeks

Week	Patients (responders/evaluable), n/N	Response Rate, %
4	4/38	11
8	9/38	24
12	12/37	32
16	14/32	44
20	10/28	36
24	10/21	48
28	9/17	53

*CT* computed tomography

## Discussion

The current study from a phase 1/2 trial provides evidence that pancreatic tumors and their associated metastases are hypermetabolic. ^18^F-FDG PET/CT is a useful tool for monitoring treatment response, changes in tumor metabolic activity, and predicting survival in this disease setting.

The results described here support the previously reported survival benefit and antitumor activity of the *nab*-paclitaxel 125 mg/m^2^ plus gemcitabine 1000 mg/m^2^ regimen for treating mPC [17, 22]. The CMR and PMR rates were numerically higher in patients in the *nab*-paclitaxel 125 mg/m^2^ cohort than in those in the *nab*-paclitaxel 100 mg/m^2^ cohort (34% vs 23% and 66% vs 54%, respectively). Furthermore, a survival difference favoring the 125 vs the 100 mg/m^2^ *nab*-paclitaxel group was observed in both the PET-evaluable population (> 4-month survival benefit for 125 vs 100 mg/m^2^ [median, 15.6 vs 10.9 months, respectively]) and the all-evaluable population (≈ 3-month survival benefit [median, 12.2 vs 9.3 months, respectively) [22]. The longer survival in patients treated with *nab*-paclitaxel 125 mg/m^2^, despite a similar or even higher baseline SUV_max_, suggests a greater clinical benefit with the 125 mg/m^2^ dose than with the 100 mg/m^2^ dose. Additionally, the high metabolic response rates observed in the PET-evaluable cohorts (100% and 77% in the 125 and 100 mg/m^2^ *nab*-paclitaxel dose cohorts, respectively) may reflect the previously published relatively high radiological response rates observed in this study (48% and 45% in the 125 and 100 mg/m^2^ *nab*-paclitaxel dose cohorts, respectively [22]).

In this study, ^18^F-FDG PET/CT responses at 6 or 12 weeks predicted survival among patients in the *nab*-paclitaxel 125 mg/m^2^ cohort. The OS in patients in this cohort who had a CMR was significantly longer than that of patients who had a PMR (median, 23.0 vs 11.2 months, respectively; *P* = 0.011)—an observation that is consistent with our previous report for all patients with a CMR vs those without a CMR (median, 20.1 vs 10.3 months, respectively; *P* = 0.01) [22]. In addition, a significant correlation was found between the best percentage of change in PET score—evaluated as a continuous variable—and survival. These findings agree with the findings of a phase 1 study of patients with advanced PC that reported a significant correlation between improved OS and a decreased metabolic activity via PET in hepatic metastases [24]. That study, by Beatty and colleagues, underscores the utility of PET in analyses involving immunotherapies, in which radiological methods by CT could be complicated by inflammation. The validation of correlating a change in metabolic activity with survival strongly supports the clinical utility of PET analysis.

The analysis of change in SUV_max_ from week 6 to week 12 is informative of the dynamics of tumor metabolic response to therapy. This analysis suggests that when patients experience a metabolic response following treatment with *nab*-paclitaxel 125 mg/m^2^ plus gemcitabine 1000 mg/m^2^, that response is unlikely to be reversed over a 6-week period. Determining the clinical activity of a particular treatment regimen at an early time point could inform the physician about whether to continue the current therapy or to switch to a different therapeutic option. Early assessment of clinical activity is particularly important given the recent interest in developing treatment plans for patients with mPC. In a study of neoadjuvant human epidermal growth factor receptor 2–positive breast cancer, metabolic responses determined by ^18^FDG PET/CT were evident at 2 weeks posttreatment, and a significant correlation (*R* ^*2*^ = 0.81) was observed between the change in SUV_max_ at weeks 2 and 6 [5]. At weeks 2 and 6, the metabolic response rates (CMR plus PMR) of the primary tumor were 72% and 60%, respectively. Reductions in tumor metabolic activity reflected treatment outcome—the reduction in SUV_max_ at both 2 and 6 weeks was significantly greater in those with a pathological complete response (pCR) vs those without a pCR (*P* = 0.02 at both time points). Similarly, in a study of patients with previously treated non-small cell lung cancer, patients with a PMR 14 days after erlotinib treatment (26% of PET-evaluable patients) had a longer OS compared with patients without a metabolic response (*P* = .03) [23].

Previously, a controversy existed among oncologists regarding the usefulness of ^18^F-FDG PET/CT for measuring tumor response in the mPC setting because of the perceived lack of sufficient FDG uptake in pancreatic lesions and the increased expense associated with the procedure. The current analysis confirms that primary pancreatic tumors and their metastases are PET avid and that ^18^F-FDG-PET/CT is useful for monitoring their metabolic activity in this setting. Although the combined/hybrid device is more expensive than CT alone, it has the advantage of providing both functions as stand-alone examinations. This allows for improved staging and monitoring of disease progression and, thus, for potentially improved treatment plans. Furthermore, our data suggest that ^18^F-FDG PET/CT is a valuable adjunct for assessing treatment response and can predict clinical outcomes in patients treated with *nab*-paclitaxel plus gemcitabine, particularly in patients who received the indicated dose and schedule.

Although the use of ^18^F-FDG PET/CT in the mPC setting is promising, our study had some limitations. First, the number of patients evaluated by ^18^F-FDG PET/CT was relatively small, and not all trial participants underwent PET/CT imaging because of either patient or physician discretion. Another limitation of the analysis was that 15 patients (6 in the *nab*-paclitaxel 125 mg/m^2^ cohort) had PET scans only at baseline. Although this limitation could have had some effect on the overall correlation of PET responses with survival, it is unlikely that all of the patients would have had a CMR if a follow-up PET scan had been performed. Such a result would not negate the important observation of the long survival durations in patients who experienced a CMR.

The PET results of the phase 1/2 study presented here agree with those of the phase 3 MPACT trial in that PET appeared to be a more sensitive modality than spiral CT for measuring treatment response [12]. Both data sets support the notion that a metabolic response by PET is associated with longer survival. It is important to point out that these were distinct studies that differed in design and conduct, as reflected by the different median baseline SUV_max_ values (6.5 in this study and 4.6 in the phase 3 trial; both among patients in the *nab*-paclitaxel 125 mg/m^2^ plus gemcitabine cohorts); thus, a direct comparison cannot be made.

## Conclusions

The results of this study support the idea that metastatic and primary pancreatic lesions are FDG avid and that ^18^F-FDG PET/CT in mPC is a useful tool for monitoring treatment response in patients treated with *nab*-paclitaxel plus gemcitabine, particularly at the indicated dose and schedule. The metabolic response rates observed in this study using PET/CT support the activity of the *nab*-paclitaxel 125 mg/m^2^ plus gemcitabine 1000 mg/m^2^ combination regimen for treating mPC. The significant correlation between the decrease in metabolic activity, as evident by FDG uptake, and decreased risk of death highlights the clinical utility of PET and suggests that this modality may be useful in predicting the success of experimental regimens. The median OS of 23.0 months in the CMR group deserves further evaluation in subsequent studies.

## Abbreviations

^18^F-FDG: [^18^F]2-fluoro-2-deoxyglucose
CA 19–9: Carbohydrate antigen 19–9
CMR: Complete metabolic response
CT: Computed tomography
ECOG PS: Eastern Cooperative Oncology Group performance status
EORTC: European Organisation for Research and Treatment of Cancer
FOLFIRINOX: Folinic acid, 5-fluorouracil, irinotecan, and oxaliplatin
mPC: Metastatic pancreatic cancer
*nab*-P: *nab*-paclitaxel
OS: Overall survival
pCR: Pathological complete response
PET: Positron emission tomography
PMD: Progressive metabolic disease
PMR: Partial metabolic response
RECIST: Response Evaluation Criteria In Solid Tumors
ROI: Region of interest
SMD: Stable metabolic disease
SUV: Standardized uptake values
SUV_max_: Maximum standardized uptake values
SUV_max_ Total: Standardized uptake values of all target lesions
